# Mitoxantrone-Surfactant Interactions: A Physicochemical Overview

**DOI:** 10.3390/molecules21101356

**Published:** 2016-10-13

**Authors:** Mirela Enache, Ana Maria Toader, Madalin Iancu Enache

**Affiliations:** 1 Institute of Physical Chemistry Ilie Murgulescu, Romanian Academy, Splaiul Independentei 202, Bucharest 060021, Romania; ancutatoader@yahoo.fr; 2 Institute of Biology Bucharest, Romanian Academy, Splaiul Independentei 296, Bucharest 060031, Romania; madalin.enache@ibiol.ro

**Keywords:** mitoxantrone, surfactants, micelles, binding constant, partition coefficient

## Abstract

Mitoxantrone is a synthetic anticancer drug used clinically in the treatment of different types of cancer. It was developed as a doxorubicin analogue in a program to find drugs with improved antitumor activity and decreased cardiotoxicity compared with the anthracyclines. As the cell membrane is the first barrier encountered by anticancer drugs before reaching the DNA sites inside the cells and as surfactant micelles are known as simple model systems for biological membranes, the drugs-surfactant interaction has been the subject of great research interest. Further, quantitative understanding of the interactions of drugs with biomimicking structures like surfactant micelles may provide helpful information for the control of physicochemical properties and bioactivities of encapsulated drugs in order to design better delivery systems with possible biomedical applications. The present review describes the physicochemical aspects of the interactions between the anticancer drug mitoxantrone and different surfactants. Mitoxantrone-micelle binding constants, partitions coefficient of the drug between aqueous and micellar phases and the corresponding Gibbs free energy for the above processes, and the probable location of drug molecules in the micelles are discussed.

## 1. Introduction

Mitoxantrone (1,4-dihydroxy-5,8-bis[2-(2-hydroxyethylamino)ethylamino]anthracene-9,10-dione) is a synthetic anthracenedione anticancer drug developed in the 1980s as a doxorubicin analogue in a program to find drugs with improved antitumor activity and decreased cardiotoxicity compared with the anthracyclines [1]. It is the only drug of the anthracenedione class approved for clinical use. Mitoxantrone is used primarily in therapy for breast cancer, acute leukemia, lymphoma and prostate cancer and, more recently, in the active forms of relapsing-remitting or secondary progressive multiple sclerosis [2,3,4]. Previous studies suggest that mitoxantrone has less cardiotoxicity than anthracyclines at equivalent doses, but further investigations revealed that mitoxantrone presents a similar cardiac toxicity clinical profile to doxorubicin and that the toxicity can occur at any time during therapy, and the risk increases with increased cumulative dose [5,6,7].

The mechanism of the mitoxantrone-associated cardiotoxicity is still poorly understood and can involve formation of reactive oxygen species [8,9,10], altered function of myocardial adrenergic receptors [11], multiple disturbances in calcium homeostasis [12], impaired expression of various important cardiac proteins [13], proteome changes including profound impairment of mitochondrial energy production, perturbations in energy channeling and impairments of mitochondrial antioxidant protection [14].

**Figure 1:**
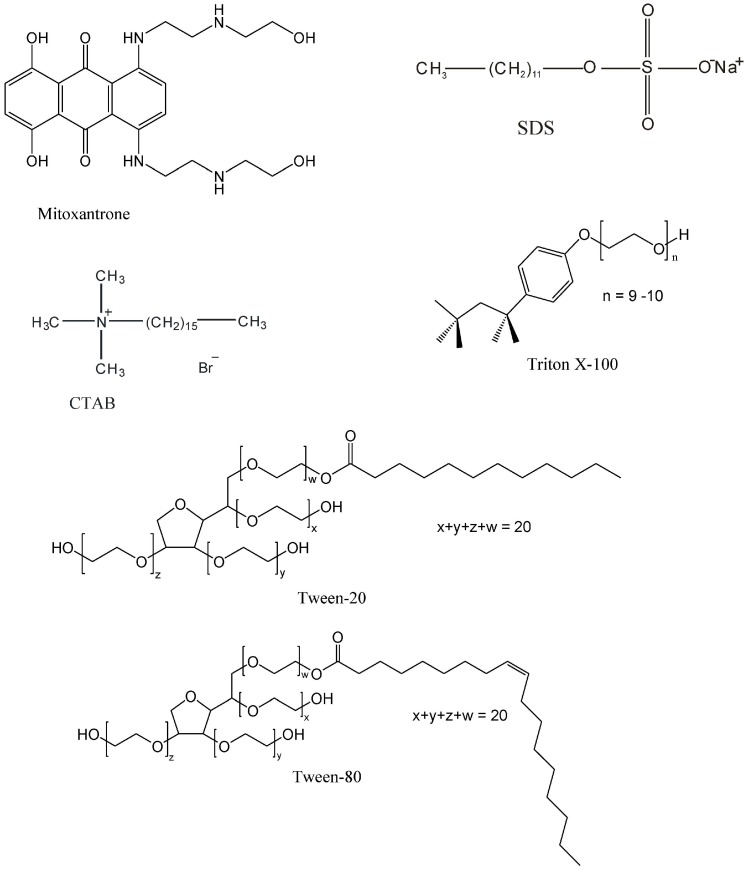
Chemical structures of mitoxantrone, SDS, CTAB, Triton X-100, Tween-20 and Tween-80.

The antitumor activity of mitoxantrone is related to its ability to bind to DNA and to inhibit both DNA replication and DNA-dependent RNA synthesis [15,16,17,18,19,20,21]. Besides, mitoxantrone is also a potent inhibitor of topoisomerase II, which is an important enzyme for the repair of damaged DNA and this results in single and double strand breaks [3,22]. Structurally mitoxantrone is symmetrical, containing a tricyclic planar chromophore substituted with two nitrogen-containing side chains (Figure 1). The planar anthraquinone ring is the key element for mitoxantrone molecule intercalation between the base pairs of DNA, whereas the basic side groups contribute to the electrostatic binding with the negatively charged DNA phosphate backbone [23].

The drug molecule has to pass through the cellular and nuclear membranes and to interact with them before approaching the target DNA inside of the cell. The exact manner in which bioactive molecules can interact and penetrate cellular membranes, and molecular details of these mechanisms are foremost to their chemotherapeutic action because the membrane acts as a barrier to the permeation of polar molecules and this effect is mainly due to the hydrophobicity of the membrane interior [24]. It is therefore interesting to see how different membrane parameters like surface charges, hydrophobic effect, and length of the fatty acid chain would affect the structure of mitoxantrone and its interaction with membranes.

Biological membranes are extremely complicated dynamic structures due to the existence of lipid domains, lipid asymmetry, coexistence of phases and diversity in lipid composition. Also, the complexity of biological membranes is further increased by their association with proteins and carbohydrates. Because of this complexity, the interactions between biological membranes and bioactive ligands are very difficult to investigate in a real situation [25]. Therefore, simplified model membranes in which the organization best mimics the bilayer lipid arrangement found in natural membranes have been developed [26,27].

Micelles with their hydrophilic surface and hydrophobic interior serve as simple membrane mimetic systems that allows controlled studies of the effect of different membrane parameters on the binding of drug molecules [28]. The use of surfactant micelles as membrane models is considered to be more advantageous, compared to liposomes and soluble polymers, because of their simplicity, low toxicity, tunable charge, narrow size distribution, longer residence time in the system and the enhanced bioavailability and stability of drug through micelle encapsulation [29,30]. Besides being used as biomembrane model systems, micelles can be used to solubilize poorly soluble drugs, thus increasing their bioavailability. Micelles are known to have an anisotropic water distribution within their structure; the water concentration decreases from the surface towards the core of the micelle, with a completely hydrophobic (water-excluded) core [31]. Consequently, the position of a solubilized drug in micelles depends on its polarity: nonpolar molecules will be solubilized in the micellar core, and drug molecules with intermediate polarity will be distributed along the surfactant molecules in certain intermediate positions [28,32].

Ionic (sodium dodecyl sulfate (SDS), cetyltrimetylammonium bromide (CTAB)) and non-ionic surfactants (Triton X-100, Brij-35 and Tweens) are commonly accepted as model systems for studying different aspects of membrane interactions with drug molecules, including their localization [33,34,35]. Because many biological processes occur at the ionizable surface of membranes or in the hydrophobic region, a comparative study of the drug interaction with different charged surfactants may provide information about the nature of the binding forces involved in the drug-membrane interaction [32,33,34,35].

The change of the pharmacological behavior of drugs by their encapsulation in micelles can be a way to improve the treatment efficacy and to overcome the toxic side effects of drugs, ensuring the transport to specific sites of action without loss. Systematic studies in this field must involve the evaluation of drug interaction with biological membranes. The main reason for this is that the nature and magnitude of drug/biomembrane interaction can determine the drug release from the carrier [36]. Different drug-delivery systems have been studied in an attempt to improve the antitumour effect of mitoxantrone and to prevent its harmful side effects [37,38,39,40].

As the surfactant micelles can be used as drug carriers and simplified model membranes in order to obtain information about the interaction with biological membranes, a systematic investigation of the interaction of mitoxantrone with anionic (SDS) [41], cationic (CTAB) [42] and non-ionic (Triton X-100, Tween-20, Tween-80) [43] surfactants has been performed. Ionic micelles were chosen as a model of the lipid bilayer in order to investigate the electrostatic contribution to the drug binding (the influence of different charges at the polar surfactant head groups), while the non-ionic micelles were chosen in order to evaluate the hydrophobic contribution to mitoxantrone binding.

This review provides an overview on these studies regarding the interaction of mitoxantrone with different surfactants. UV-Vis absorption spectroscopy has been used to evaluate the parameters such as binding constants, partition coefficients, free energy of interaction and also to predict the location of drug molecules in the micelles. Quantitative understanding of the drug-micelle interactions is an important step in the design of efficient drug delivery systems.

## 2. UV-Vis Absorption Studies

The surface of biological membranes frequently presents a net charge due to the ionisable head groups of lipids. Therefore, the binding characteristics of charged and uncharged drug molecules may be very different [44]. Surfactant micelles bearing different charges on their surface can be used in UV-Vis absorption spectroscopy studies in order to obtain qualitative and quantitative information about drug-micelle interactions.

Mitoxantrone is a weakly basic drug with two ionizable amine groups (pKa values of 8.3–8.6) [45] and its distribution will be influenced by the micro-environmental pH. pKa shifts were observed for different drugs upon their binding to micelles or bilayers [32,36,44,46,47]. The pH-dependent behaviour of the mitoxantrone-surfactant interaction was evaluated in vitro at pH 7.4, when mitoxantrone is a dication with two positive charges on the nitrogen atoms from the side chains [19] and pH 10, when it is uncharged due to the deprotonation of the side chain amino groups [48].

The visible absorption spectrum of mitoxantrone in phosphate buffer pH 7.4 consists of three overlapping spectral absorption bands: two absorption bands at 660 and 610 nm, and a shoulder at about 570 nm, more evident at higher drug concentrations. The shape of the absorption spectrum of mitoxantrone is dependent on concentration and, as this dependence is usually assigned to the formation of molecular aggregates, the band at 660 nm was assigned to the monomer (M), the band at 610 nm to the dimer (D) and the band around 560 nm to the formation of the higher aggregates of the drug [49]. In carbonate buffer pH 10, the absorption maxima of dimer and monomer are red shifted and the ratio of monomer to dimer absorbances decreases, indicating that the dimerization process is favored in a basic environment (Table 1) [49]. Also, at both pH values, the positions of the absorption maxima of dimer and monomer do not change with temperature, but the ratio of monomer to dimer absorbance increases with temperature, indicating the dissociation of mitoxantrone aggregates (dimers or higher aggregates) with increasing temperatures [50].

The absorption spectral behavior of mitoxantrone shows a strong dependence on surfactant type and concentration. In the case of SDS [41], the intensity of both the monomer and dimer absorption bands decreases for surfactant concentrations lower than the critical micellar concentration (CMC). When the SDS concentration is lower than the CMC, the cationic mitoxantrone molecules are strongly attracted by the anionic SDS molecules and the decrease of absorbance was assigned to the neutralization of mitoxantrone charges by electrostatic interaction between the positively charged amino groups of the drug and the negatively charged surfactant groups, allowing the formation of a drug–SDS ion-association complex. The electrochemical results [41] and Job’s method of continuous variation and molar ratio method [51] indicated that the stoichiometry of the mitoxantrone-SDS complex is 1:2. Due to the neutralization of the charges of the mitoxantrone dication in the ion-association complex, the mitoxantrone molecule becomes more hydrophobic and its dimerization increases [41]. 

Unlike the anionic surfactant SDS, the presence of premicellar CTAB and non-ionic surfactants concentrations do not change the absorption spectrum of mitoxantrone [42,43]. For surfactant concentrations higher than the CMC, the intensity of both monomer and dimer bands increases, but the monomer absorbance at 660 nm becomes predominant. Also, for micellar surfactant concentrations both absorbance maxima are red shifted (Table 1). This bathochromic shift indicates the interaction between mitoxantrone and surfactant micelles and the transfer of mitoxantrone molecules are from the highly polar aqueous phase into a relatively nonpolar micellar environment. The increase in the absorption maxima at 660 nm with the increase of surfactant concentration above CMC is due to the interaction of mitoxantrone with micelles and this interaction induces the dissociation of the mitoxantrone aggregates. Thus, mitoxantrone molecules are encapsulated in micelles as monomer, following the equilibria: mitoxantrone aggregates ↔ mitoxantrone monomer (bulk) ↔ mitoxantrone monomer (micelles), similar to the pinacyanol cationic dye [52,53]. Therefore, the presence of micelles causes a shift this equilibrium to the right and the conversion of mitoxantrone aggregates to monomer and encapsulation of mitoxantrone monomers in micelles.

**Figure 2:**
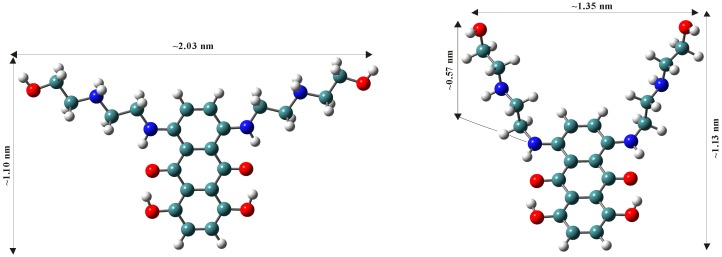
Optimized geometries of mitoxantrone monomer in neutral form (**right**) and dication form (**left**).

Theoretical calculations were carried out with the Gaussian 03 software package [54] using the B3LYP/6-311G* basis set in order to predict the molecular structure of mitoxantrone monomer and dimer. Water solvent effects were simulated by the conductor-like polarizable continuum model (CPCM) [55]. Optimized geometries of monomer mitoxantrone in neutral and dication form are presented in Figure 2. When the mitoxantrone monomer has two positive charges on the side chain nitrogen atoms (like in the experimental conditions at pH 7.4), the molecule has both side chains almost in the same plane as the aromatic rings (the dihedral angle between them being 178 degrees) and the highest distance between two side chains is 2.03 nm. When mitoxantrone monomer is not charged (like in experimental conditions at pH 10), the molecule does not have a fully planar structure, the amino alkyl side chains are entirely out of plane, with a 81 degrees out of planarity of the whole geometry, close to the 77 degrees from literature data [56]. The two side chains are closer to each other, the distance between them being 1.35 nm. 

**Figure 3:**
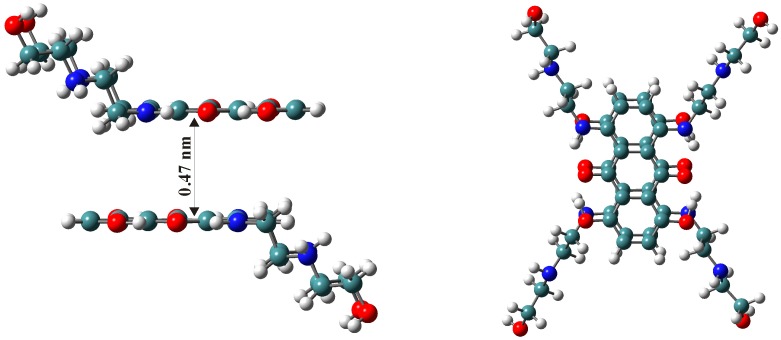
Optimized geometry of mitoxantrone dimer: the side view (**right**) and the perpendicular to the planes of the chromophores view (**left**).

In the mitoxantrone dimer geometrical arrangement (Figure 3), the planes of the drug chromophores are parallel to each other due to π-π stacking interactions and situated at 0.47 nm apart and this value is close to the distance between two pyrene molecules (0.42 nm) [57]. This geometric arrangement of mitoxantrone dimer in solution resembles the calculated structures proposed using NMR analysis [58].

Because a typical micelle has a diameter about 5 nm [59] and due to the antiparallel orientation of alkyl side chains in the dimer geometry, it is difficult for mitoxantrone dimer to be entrapped in micelles, thus we assumed that mitoxantrone is encapsulated in micelles in monomer form.

**Figure 4:**
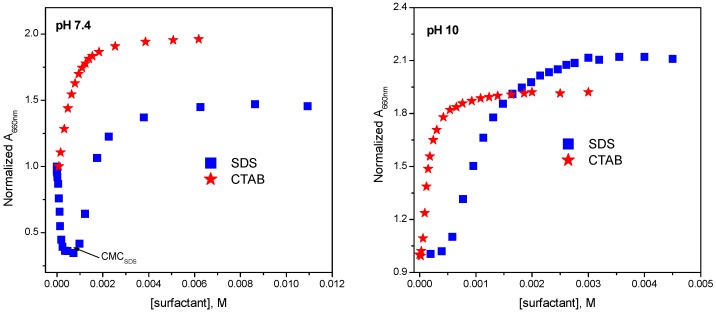
Variation of the monomer normalized absorbance at 660 nm with SDS and CTAB concentration at pH 7.4 and pH 10. (The figure was drawn based on references [41,42]).

The CMC values for all surfactants in the presence of mitoxantrone were determined from the change in the absorption spectrum of mitoxantrone and are smaller than the values in pure water, knowing that the CMC is influenced by the presence of different ions and molecules [60,61].

when mitoxantrone monomers are encapsulated in micelles [42,43]. The variation of absorbance at 660 nm as a function of SDS and CTAB concentration at pH 7.4 and 10 is presented in Figure 4. In the case of SDS two distinct processes depending on the surfactant concentration are observed at pH 7.4: process I in premicellar range, assigned to the electrostatic interaction between positively charged mitoxantrone molecules and negatively charged SDS monomers and process II in micellar surfactant concentrations, when the SDS micelles are formed and the drug is encapsulated in micelles in monomer form [41]. For CTAB, Triton X-100, Tween-20 and Tween-80 surfactants only one process is observed for micellar surfactant concentration

Erdinc et al. studied the interaction of the cationic anthracycline drug epirubicin with anionic (SDS), cationic (CTAB) and non-ionic (Triton X-100, Tween-20) surfactants by using UV-Vis absorption spectroscopy [61]. Their results indicated that in the case of SDS, epirubicin-SDS molecular complex formation takes place at premicellar surfactant concentrations due to electrostatic interactions followed by dissociation and further incorporation at micellar concentrations. Also, it was found that the binding constant is higher for non-ionic surfactants than SDS and a very week interaction occurred between epirubicin and CTAB due to electrostatic repulsion [61].

Zafar and co-workers reported the interaction of some anticancer uracil derivatives with SDS and CTAB surfactants [62]. Cyclic voltammetry and UV-Vis spectroscopic techniques were used to evaluate the binding constants, partition coefficients between bulk and micellar phase, and the number of drug molecules incorporated per micelle. These studies revealed that at premicellar concentrations, the binding is mainly due to the electrostatic interactions between the surfactant monomers and the drug molecules, while in the postmicellar region, drug is encapsulated into micelles due to electrostatic as well as hydrophobic interactions [62].

Studies performed by Bhattacharjee et al. [63] indicated that the ionic mixed micelles of Tween-80-NaDC (sodium deoxycholate) can encapsulate the cationic drug doxorubicin by noncovalent electrostatic interaction and doxorubicin encapsulated in these mixed micelles has greater anticancer activity in different cancer cell lines as compared to doxorubicin solution. Also, the electrostatic binding between doxorubicin and anionic surfactant aerosol OT leads to the formation of a hydrophobic drug-surfactant complex and this complex can be encapsulated in Pluronic block copolymer (P123) micelles without disrupting the structure of aggregates [64].

Cyclic voltammetry investigation of the interaction between doxorubicin and SDS outlined a weak predominantly electrostatic drug-surfactant interaction in the range of pre-micellar and micellar concentrations of surfactant [65].

Spectral studies regarding the interaction of 2-amino-3-hydroxyanthraquinone (AQ), an analogue of the anthracycline anticancer drugs, with SDS and CTAB showed that the hydrophobic interactions play a crucial role in the binding of AQ to SDS micelles, while the hydrophilic interactions plays an important role in its interaction with CTAB micelles [66]. 

Spectral and electrochemical investigation of the interaction of anticancer drug actinomycin D (actinomycin D contains a 2-aminophenoxazin-3-one chromophore and two cyclic pentapetide lactones) with different surfactants indicated significant differences in the strength of the interaction, the binding constants being higher for charged surfactants (SDS, CTAB) than non-ionic surfactants (Triton X-100) [67,68]. Also, the binding constants for the interaction of actinomycin D with CTAB, SDS and Triton X-100 are much smaller than the binding constants for the interaction of mitoxantrone with these surfactants.

**Figure 5:**
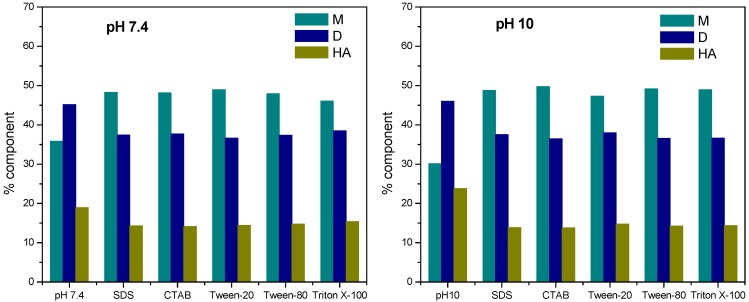
Variation of the percent of component band areas in deconvoluted spectra for monomer (M), dimer (D) and higher aggregates (HA) of mitoxantrone in phosphate buffer pH 7.4, carbonate buffer pH 10 and micellar concentrations of SDS (8.64 × 10^−3^ M), CTAB (3.88 × 10^−3^ M), Tween-20 (3.12 × 10^−2^ M), Tween-80 (3.46 × 10^−2^ M) and Triton X-100 (2.68 × 10^−2^ M).

In order to quantify the evolution of the three overlapping absorption components of mitoxantrone in different conditions, the deconvolution of the spectra in elementary bands was performed using the Gaussian multi-peaks function in the PeakFit 4.11 software. The goodness of the fit was considered from the fitting parameter (R^2^~1) and the symmetrical distribution of the residuals. Figure 5 shows the variation of monomer, dimer and higher aggregate components of mitoxantrone in phosphate buffer pH 7.4, carbonate buffer pH 10 and in the presence of micellar concentrations of SDS (8.64 × 10^−3^ M), CTAB (3.88 × 10^−3^ M), Tween-20 (3.12 × 10^−2^ M), Tween-80 (3.46 × 10^−2^ M) and Triton X-100 (2.68 × 10^−2^ M), obtained from deconvolution of the spectra. 

It can be observed that for both pH values, the dimer component percent is almost constant (about 45%), but at pH 10 the higher aggregates component increases on expense of monomers, indicating that the aggregation of mitoxantrone monomers is favored in basic medium and dimers and higher aggregates are the predominant species. For pH 7.4 and 10 and micellar concentrations of SDS, CTAB, Tween-20, Tween-80 and Triton X-100, the monomer and dimer components increases on expense of higher aggregate component, indicating the dissociation of these species caused by the interaction of mitoxantrone with surfactant micelles (monomer ↔ dimer ↔ higher aggregate equilibrium is shifted to the monomer and dimer formation). It can also be observed that for both pH values, the presence of anionic, cationic and non-ionic surfactant micelles induces the disaggregation of mitoxantrone and the percent of monomer, dimer and higher aggregate components is almost the same for all investigated surfactants.

The interaction of mitoxantrone with surfactant micelles induces the dissociation of drug aggregates similar with the interaction of mitoxantrone with DNA [69]. This similar behavior can be explained by analogy between DNA and SDS micelles (negatively charged surface and hydrophobic interior) as receptor for intercalating drugs. Digman and co-workers investigated the interaction of intercalating drug daunomycin with surfactant micelles as a model for the hydrophobic contribution to the free energy of DNA intercalation reactions [70].

Absorption and circular dichroism spectroscopy, and thermal denaturation studies have shown that the presence of SDS micelles induces the exclusion of intercalated mitoxantrone monomer from DNA and further their encapsulation in micelles [69]. This deintercalation process of intercalated drug molecules from DNA in the presence of surfactant micelles and their transfer inside of micelles was observed for different ligands [71,72,73,74,75,76]. Considering that amphiphilic molecules are present in real biological systems (i.e., phospholipids in the cell membranes, polyamines in nucleus, bile salts in the bile), this deintercalation process induced by micelles may have significant importance in biological processes [71].

## 3. Polarity of the Micellar Environment and Probable Location of Mitoxantrone

A micellar structure is characterized by different layers: (a) the hydrophobic core containing the hydrocarbon tails of the surfactant molecules; (b) Stern layer containing compact head groups of ionic surfactants or the palisade layer composed of the polyoxyethylene chains of non-ionic surfactants; and (c) the surface of micelles [31,77]. A drug molecule can interact with micelles in different ways, depending on the drug hydrophobicity: hydrophilic drugs can be adsorbed on the surface of the micelle, hydrophobic molecules can be trapped in the hydrophobic core of the micelles or, in the case of drugs with intermediate solubility should be located in intermediate positions within the micelle such as between the hydrophilic head groups of non-ionic micelles and in the palisade layer between the hydrophilic groups and the first few carbon atoms of the hydrophobic groups [31,77]. The location of molecules into micelles determines the extent of solubilization, the pharmacokinetics and biodistribution of incorporated drug molecules [77].

As seen in Table 1, the presence of micellar concentrations of SDS, CTAB, Triton X-100, Tween-20 and Tween-80 is associated with a red shift of monomer absorption maxima at both pH values, suggesting that the micro-environment around mitoxantrone molecule is perturbed in comparison with phosphate buffer pH 7.4 and carbonate buffer pH 10. At pH 7.4, the red shift increases with surfactant in the order CTAB > Tween-80 > Tween-20 > Triton X-100 > SDS, whereas at pH 10 the red shift follow the order CTAB > Tween-80 = Tween-20 = Triton X-100 > SDS. This red shift indicates the transfer of mitoxantrone molecules from polar phase to a less polar phase in micellar medium. The octanol:water partition coefficient of mitoxantrone at pH 7.4 is logP = 0.79, which indicates that mitoxantrone is a fairly lipophilic drug [78]. Therefore, in the presence of surfactant micelles mitoxantrone molecules prefer to move from polar aqueous medium in more hydrophobic medium of micelles.

**Figure 6:**
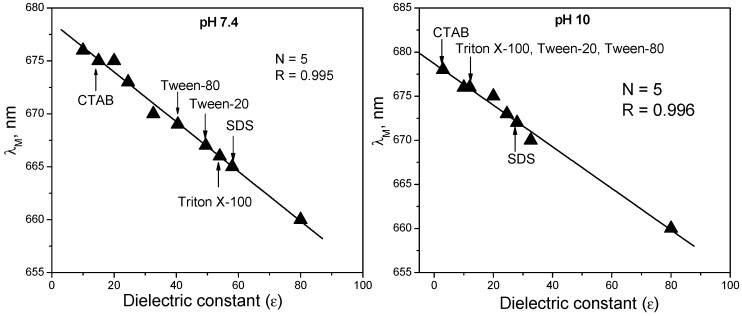
Absorption maxima of mitoxantrone monomer (λ_M_) in different solvents (water, methanol, ethanol, propanol, *tert*-butanol) as a function of the dielectric constant at pH 7.4 and 10. The positions for the surfactant micelless at pH 7.4 and 10 are shown on the graphs (the figure was drawn based on the references [41,42,43]).

Information about the position of mitoxantrone molecule in the micelle was obtained by comparing the absorption spectra of mitoxantrone in the presence of surfactant micelles with the spectra in water and solvents of different polarities. The absorption spectra of mitoxantrone recorded in protic solvents with different polarities indicated a red shift of both absorption maxima with the reduction of the solvent polarity. Also, the relationship between the position of monomer absorption maximum and the dielectric constant is linear at both pH values (Figure 6).

Generally, these spectral shifts are interpreted as polarity changes of the immediate vicinity of the drug molecule [79]. Therefore, the substitution of corresponding absorption maxima of monomer band for CTAB, SDS, Triton X-100, Tween-20 and Tween-80 micelles allowed one to determine polarity values corresponding to effective dielectric constants of 20, 58, 54, 49.5 and 40.5, respectively, for pH 7.4. For pH 10, the following effective dielectric constants were obtained: 3 for CTAB, 28 for SDS and 12 for Triton X-100, Tween-20 and Tween-80. It can be observed that for all surfactants, the dielectric constant at pH 10 is smaller than that at pH 7.4, indicating that at pH 10 when mitoxantrone molecule becomes uncharged and more hydrophobic penetrates deeper into micelles. 

Since the micelle interface is an environment with a dielectric constant (ε = 36) [80] intermediate between water (ε = 80) and 1,4-dioxane (ε = 2), mitoxantrone is encapsulated in CTAB and SDS micelles as monomer, and most probably situated in the micelle surface layer.

In the case of SDS micelles, the mitoxantrone chromophore ring is oriented towards the micelle core and both positively charged side chains oriented towards the negatively sulfate groups of SDS, both polar and electrostatic interactions playing important role in the drug-micelle binding [41]. Also, this electrostatic attraction between opposite charges on mitoxantrone and SDS does not let the drug molecules penetrate deeply into the micelles. Therefore, the drug molecules are solubilized near the micelle surface.

In the case of CTAB micelles, the location of mitoxantrone molecules in the micelle surface layer can be explained by a cation-π interaction between the uncharged ring systems of mitoxantrone and the cationic head groups of CTAB, similar to the cationic dye pinacyanol [42,79]. The lower spectral shift corresponding to a higher dielectric constant observed for the SDS micelles indicates that the micropolarity around mitoxantrone molecules in SDS micelles is different from that in CTAB micelles. In the case of positively charged CTAB micelles the electrostatic interactions between mitoxantrone molecules and micelles are absent and the hydrophobic effect prevails, resulting in a deeper penetration of mitoxantrone molecule into micelles (a higher spectral shift is observed, correlated with smaller dielectric constant). The flexibility of longer aliphatic chains of CTAB compared with SDS micelles can favor the easier movement of mitoxantrone molecules toward the core of CTAB micelles, resulting in a higher spectral shift [42,81].

In the presence of non-ionic micelles, the monomer band is split into two components (spectra in Figure 2, [43]), which was not observed for SDS and CTAB micelles, but is more evident in the drug spectrum of mitoxantrone in 1,4-dioxane. Therefore, the new band around 647 nm, increasing with surfactant concentration, was assigned to the ionic-hydrophobic interactions of a part of the drug molecule present in a more hydrophobic medium [43]. The palisade layer composed from the polyoxyethylene chains has a dielectric constant of 40–50 [82] and the major part (band at 660 nm) of the drug molecule is most probably located in the palisade layer, which is known to be much thicker (25 Å) than the Stern layer of ionic micelles (6–9 Å) [42,83], similar to the case of the antitumor antibiotic epirubicin [61].

## 4. Binding Parameters

Better understanding of drug-micelle interaction is achieved by both explaining the nature of interaction and by quantifying its magnitude through the determination of binding constant and/or partition coefficient. Mitoxantrone-micelles binding constants, partition coefficients of drug between aqueous and micellar phases and the corresponding Gibbs free energy for both processes were evaluated from the changes in spectral characteristics of mitoxantrone in the presence of different surfactant micelles.

### 4.1. Binding Constant

The mitoxantrone-surfactant micelles binding constants (K_b_) were evaluated from the values of the absorbance of the monomer band assuming a 1:1 interaction between the drug and the surfactant micelles:

(1) $$D+M\overset{K_{b}}{⟷}\mathrm{DM}$$

where D, M and DM represent the drug, micelle and drug-micelle complex. The binding constant is given by:

(2) $$K_{b}=\frac{C_{b}}{\left(C_{T}-C_{b}\right)([\mathrm{surfactant}]-C_{b})}$$

where C_T_ is the total concentration of mitoxantrone and C_b_ is the concentration of the bound mitoxantrone. The absorbance of the solution at a wavelength in the band of mitoxantrone, where the surfactant is supposed not to absorb, is given by:

(3) $$A=\varepsilon_{f}C_{f}+\varepsilon_{b}C_{b}$$

where C_f_, ɛ_f_ and C_b_, ɛ_b_ are the concentrations and the molar absorption coefficients of the free and bound mitoxantrone, respectively. If the concentration of the bound mitoxantrone is smaller than the mitoxantrone initial concentration, the following formula is obtained:

(4) $$A=\frac{A_{0}+A_{b}K_{b}[\mathrm{surfactant}]}{1+K_{b}[\mathrm{surfactant}]}$$

where A is the measured absorbance, A_0_ is the absorbance of mitoxantrone in the absence of surfactant and A_b_ is the absorbance of mitoxantrone bound to surfactant micelles. Nonlinear regression using Euqation (4) [84] allows determining the binding constant of mitoxantrone to surfactant micelles. The results are presented in Table 2. The Gibbs free energy of binding of mitoxantrone to surfactant micelles can be obtained by the following equation:

∆G_b_^0^ = −RTlnK_b_ (5)

where R is the gas constant and T the absolute temperature.

Analysis of the data from Table 2 indicates that the binding constant for the interaction of mitoxantrone with all investigated micelles at pH 10 is higher than that for pH 7.4. The binding of mitoxantrone to CTAB micelles is sensitive to the charge state of the drug: the coulombic repulsion between positively charged mitoxantrone at pH 7.4 and micelle cationic head group leads to a decrease of binding constant in comparison with pH 10 [42].

In the case of neutral surfactant micelles, the binding is expected to be dominated by hydrophobic interactions. On changing the surfactant, at pH 7.4 and pH 10, the binding constant values follow the order: CTAB > SDS > Tween-80 > Tween-20 > Triton X-100. Taking into account the charges of the mitoxantrone molecule and surfactant micelles, the interaction between the cationic drug mitoxantrone and anionic SDS micelles is expected to be stronger than the interaction between cationic drug and cationic CTAB micelles because of the presence of both electrostatic and hydrophobic interaction forces [42]. However, the interaction of mitoxantrone with CTAB micelles is about 2.6 times stronger than the interaction with SDS micelles, indicating that the hydrophobic interactions have a major role in the binding of mitoxantrone to surfactant micelles [42]. This stronger interaction of mitoxantrone with CTAB micelles compared with SDS micelles can be explained by the cation-π interaction between the uncharged ring system of the drug and the cationic head groups of CTAB, which is sufficiently intense to overcome the coulombic repulsion between positively charged species [42,79].

The binding constants for the interaction of mitoxantrone with non-ionic surfactant micelles are much lower than the binding constants for the SDS and CTAB micelles at both pH values. As seen from Table 2, all ∆G_b_ values are negative, indicating that the binding of mitoxantrone monomers to surfactant micelles occurs spontaneously. 

### 4.2. Partition Coefficient

Partitioning of drugs between water and micellar pseudo-phase is quantitatively characterized by the partition coefficient (K_x_). It is the ratio of concentration of drug molecules in the micelle to that in bulk aqueous solution. The partition coefficient parameter is important not only in elucidating the mechanism of solubilisation, but also helps to understand how a drug is partitioned through biological membranes within the living body [85]. The partition coefficient for mitoxantrone between aqueous and micellar phases is defined according with pseudo-phase model [86,87] as:

(6) $$K_{x}=\frac{X_{\mathrm{mito}}^{m}}{X_{\mathrm{mito}}^{\mathrm{aq}}}$$

where $X_{\mathrm{mito}}^{m}$ and $X_{\mathrm{mito}}^{\mathrm{aq}}$ are the mole fractions of mitoxantrone in micellar and aqueous phase, respectively. They are related the mole fractions of mitoxantrone in micellar and aqueous phase, respectively. They are related with concentrations of species in the solubilization system:

(7) $$X_{\mathrm{mito}}^{m}=\frac{C_{\mathrm{mito}}^{m}}{C_{\mathrm{mito}}^{m}+C_{\mathrm{surfactant}}^{m}};\;X_{\mathrm{mito}}^{\mathrm{aq}}=\frac{C_{\mathrm{mito}}^{\mathrm{aq}}}{C_{\mathrm{mito}}^{\mathrm{aq}}+C_{\mathrm{surfactant}}^{\mathrm{aq}}+n_{w}}\approx \frac{C_{\mathrm{mito}}^{\mathrm{aq}}}{n_{w}}$$

where n_w_ = 55.5 M is the molarity of water, $C_{\mathrm{surfactant}}^{\mathrm{aq}}$ and $C_{\mathrm{surfactant}}^{m}$ represent concentrations of surfactant in monomeric and micellar states, respectively.

The fraction (j) of the amount of solubilized mitoxantrone is defined as:

(8) $$j=\frac{C_{\mathrm{mito}}^{m}}{C_{T}}$$

Below the CMC, this fraction j is equal to zero and increases with increasing surfactant concentration above the CMC. The fraction j can be directly calculated from the experimental data as:

(9) $$j=\frac{\Delta A}{{\Delta A}_{\infty }}$$

where ∆A = A − A_0_, ∆A_∞_ = A_b_ − A_0_. Using Equations (7) and (8), Equation (5) can be written in linear form as:

(10) $$\frac{1}{\Delta A}=\frac{1}{{\Delta A}_{\infty }}+\frac{n_{w}}{K_{x}{\Delta A}_{\infty }([\mathrm{surfactant}]+C_{T}-\mathrm{CMC})}$$

The value of K_x_ is obtained from the slope of the plot of 1/∆A versus 1/([surfactant] + C_T_ − CMC). This relation is linear for very high surfactant concentrations region below which the curve tends to bend upwards with decreasing surfactant concentration [87].

A large positive value of K_x_ indicates a higher drug concentration in micelles than in the surrounding aqueous medium, so mitoxantrone molecules move from the aqueous environment to micelles very easily. By comparing the partition coefficients (Table 3) obtained for the distribution of mitoxantrone molecules between aqueous and micellar phases, it can be observed that the uncharged mitoxantrone molecule (pH 10) exhibits a larger partition coefficient than the positively charged mitoxantrone (pH 7.4). This is due to the fact that the uncharged mitoxantrone is more hydrophobic and is better incorporated into the hydrophobic environment of the micelles than the cationically charged mitoxantrone. Theoretical calculations indicated that uncharged mitoxantrone molecule has lower dimension (1.35 nm) than charged mitoxantrone molecule (2.03 nm) and this can explain the larger partition coefficient observed for pH 10. Also, the values of K_x_ are slightly higher for CTAB than SDS micelles, indicating that the hydrophobic interactions have a major role in the distribution of mitoxantrone between micelle/water phases because of the lower polarity and longer aliphatic chains of CTAB molecules. In the case of non-ionic micelles, the values of K_x_ at pH 7.4 and pH 10 follow the order: Triton X-100 > Tween-80 > Tween-20 [43]. The higher micellar partition coefficient of Triton X-100 than Tweens at both pH values can be related to the higher aggregation number of Triton X-100 (140) [88] than Tweens (around 60) [89,90], which is responsible for higher Triton X-100 micelles which are able to accommodate more drug molecules [43]. 

The ∆G_x_ values are negative for all surfactants indicating that the partition process of mitoxantrone monomers between the micellar and the bulk water phases occur spontaneously. The value of the free energy of partition becomes more negative at pH 10 indicating that uncharged more hydrophobic mitoxantrone molecule penetrate easier into micelles. The transfer of drug molecules from aqueous phase to organic micellar phase provides a model to predict the passage of drug molecules across biological membranes.

## 5. Conclusions

Drug molecules have to pass through the cellular and nuclear membranes before reaching their DNA targets inside cancer cells. Because biological membranes are extremely complex multicomponent structures, surfactant micelles with much less complexity have been used as model systems for biomembranes and the physicochemical interactions of drugs with micelles can be visualized as an approximation for drug-membrane interactions. The detailed insights on the nature of interactions between drugs and surfactants can be obtained by using different spectroscopic techniques. The UV-Vis absorption spectroscopy technique helps evaluate the interaction parameters such as binding constants, number of binding sites, partition coefficients, free energy of binding, and also in predicting the location of drugs in the micelles.

Besides the quantitative characterization of the interaction of mitoxantrone with anionic (SDS), cationic (CTAB) and non-ionic surfactants (Triton X-100, Tween-20 and Tween-80), the investigations have indicated that the presence of surfactant micelles induces the disaggregation of mitoxantrone aggregates to monomers and mitoxantrone is encapsulated in micelles in monomeric form. Encapsulation of drug molecules in micelles in monomer form can be clinically relevant taking into account that the dose of antitumor drugs used therapeutically is generally more than tens of micromolar [91] and at these concentrations drug aggregation occurs, affecting transport across bilayer lipid membrane and consequently influencing the antitumor action [92]. Also, the drug aggregation can be associated with local toxicity and decreased bioavailability because of excessive drug accumulation at the target sites.

Better understanding of the strength and nature of drugs interaction with micellar media is not only important in the elucidation of the interactions of drugs with biological membranes but could also serve to design molecules with tailored functionalities for drug delivery development. The disaggregation efficiency and the biocompatibility of these surfactants make them an attractive choice for potential delivery systems for the anticancer drug mitoxantrone.

## Figures and Tables

**Table 1:** Absorption maxima of dimer—λ_D_, and monomer—λ_M_ of mitoxantrone in the absence and the presence of micellar solutions of CTAB, SDS, Triton X-100, Tween-20, and Tween-80, at pH 7.4 and 10.

Mitoxantrone	pH 7.4		pH 10		References
	λ_D_	λ_M_	λ_D_	λ_M_	
Buffer	610	660	614	666	[45]
CTAB	623	675	624	678	[42]
SDS	614	665	619	672	[41]
Triton X-100	615	666	623	676	[43]
Tween-20	617	667	624	676	[43]
Tween-80	618	669	624	676	[43]

**Table 2:** Binding constant (K_b_) and the Gibbs free energy of binding (∆G_b_^0^) for the interaction of mitoxantrone with different surfactant micelles.

Surfactant	pH 7.4		pH 10		References
	K_b_/M^−1^	∆G_b_^0^/kJ/mol	K_b_/M^−1^	∆G_b_^0^/kJ/mol	
CTAB	2933	−19.78	4365	−20.76	[42]
SDS	982	−16.77	1193	−17.25	[41]
Triton X-100	30	−8.52	472	−15.25	[43]
Tween-20	52	−9.79	610	−15.89	[43]
Tween-80	71	−10.56	798	−16.56	[43]

**Table 3:** Partition coefficients (K_x_) and the standard free energy change for the transfer of mitoxantrone from bulk water to micellar phase (ΔG_x_^0^) for the interaction of mitoxantrone with different surfactant micelles.

Surfactant	pH 7.4		pH 10		References
	K_x_	∆G_x_^0^/kJ/mol	K_x_	∆G_x_^0^/kJ/mol	
CTAB	1.72 × 10^5^	−29.86	2.65 × 10^5^	−30.93	[42]
SDS	4.79 × 10^4^	−26.24	6.98 × 10^4^	−27.16	[41]
Triton X-100	8.31 × 10^3^	−22.34	1.33 × 10^5^	−29.22	[43]
Tween-20	1.73 × 10^3^	−18.40	3.64 × 10^4^	−26.01	[43]
Tween-80	3.11 × 10^3^	−19.91	5.61 × 10^4^	−27.08	[43]
